# Report of the Committee of the New York State Medical Society Concerning the National Medical Convention

**Published:** 1846-04

**Authors:** 


*Report of the Committee of the New York State Medical Society concerning the National Medical Convention.*—The Committee appointed at the last Annual Meeting of the New York State Medical Society, to carry into effect a resolution passed at the same meeting inviting a National Medical Convention, in May next, would respectfully report as follows:—

In the early part of last season the Committee, through their chairman, addressed a circular containing the preamble and resolutions of the society, with such comments as were deemed advisable, to all the State medical societies and medical Colleges in the United States, as far as the existence of such societies and colleges could be ascertained. And in those States where neither State societies nor colleges existed, a circular was addressed to some leading member of the profession, inviting him to take measures for having his State represented in the proposed convention. Replies to these circulars and letters have been received from the following officers of medical societies and colleges, and private members of the profession, viz:

Drs. W. W. Morris, of Dover, Delaware State; A. H. Buchanan, of Tennessee; W. P. Johnston, of Washington city; T. T. Hewson, R. M. Huston, and W. E. Thorne, of Philadelphia; Luther Ticknor, Connecticut; W. H. McKee, of N. Carolina; E. H. Pearle, of Hanover; Paul F. Eve, of Georgia; J. H. Thompson, of New Jersey; J. W. Davis, of Indiana; A. Twitchell, of New Hampshire; John W. Draper, A. H. Stevens, Willard Parker, and C. A. Lee, of New York; J. Drake, of Ohio; Lawson, of Kentucky, and Carpenter, of Louisiana.

Delegates have been freely pledged from societies and colleges in Maine, New Hampshire, Connecticut, New Jersey, Delaware, District of Columbia, South Carolina, Georgia, Mississippi, Louisiana, Tennessee, Kentucky, Ohio, Indiana, and New York. The medical schools of Philadelphia are the only ones from which replies have been received, that decline sending delegates, and giving a hearty support to the proposed measure. Nearly every medical journal throughout the whole Union has not only favorably noticed, but warmly commended the holding of such a convention.

There are some institutions from whom no replies have been received; but, from information obtained from other sources, there is good reason for believing that most if not all these will appoint delegates as soon as they are fully assured that the convention *will* be held. It will thus be seen that in far the larger part of our Union, the invitation of this society has met with a prompt and hearty response from the profession; and it is with much regret that we find even a few institutions declining to take any part in so important a movement. But when we consider the wide extent of our territory, and the great number of our institutions, all engaged, we should hope, in a generous rivalry with each other, the expression in favor of a convention is certainly more unanimous and more promising of good than could have been anticipated. Indeed the leading and influential members of the medical profession have long felt the necessity of some national action; some central point of influence around which the active and choice spirits of the whole profession can rally, and from which may be made radiate an elevating, healthful, and nationalizing influence over the whole country.

Hence, it only remains for this society to carry out the work it has so nobly commenced. The faculty in the New York University have very generously tendered to this society, the use of any room or rooms in their college edifice, that may be desired, for the convention to meet in. Some State societies and colleges have already appointed their delegates, while others have expressed a desire that the society would fix the number of delegates to be appointed by each society and college. For the purpose of furthering the objects in view, your committee would respectfully submit the following resolutions, viz:—

1st. *Resolved*, That the preamble and resolutions passed by this society, at its annual session, Feb. 6th, 1845, did not contemplate the appointment of delegates to the National Convention, by county or merely local societies, in those States where delegates *are* appointed by a regularly organized *State* society.

2d. *Resolved*, That as some societies and colleges have already appointed their delegates, therefore, the number to be appointed by any one of these institutions should be left entirely to the discretion of the appointing body.

3d. *Resolved*, That sixteen delegates be appointed to represent this society in the proposed National Convention, viz: two from each senatorial district in the State.

4th. *Resolved*, That this society accept the offer of the faculty in the New York University, respecting their rooms; and consequently that the members of said National Convention are invited to assemble in the college edifice of the New York University, at 10 o'clock, A.M., on the *first Tuesday* in May next.

5th. *Resolved*, That a committee of three be appointed to continue the efforts to further the objects of the proposed convention; and to exert all their influence to make it truly *National* in composition and action.

6th. *Resolved*, That the above Committee be authorized to invite the superintendents of lunatic asylums in the several States to attend the Convention.

7th. *Resolved*, That the committee be further authorized and enjoined to send a copy of this report, together with the action of this society thereon, to all the medical journals in the United States, with a request that they publish the same, on or before the 1st of April next.

W. S. Davis,<br>James McNaughton,<br>Peter Van Buren, } Committee.

Albany, Feb. 3, 2846.

*New York Jour. Med.*

